# Public health round-up

**DOI:** 10.2471/BLT.25.011125

**Published:** 2025-11-01

**100 countries committed to climate action for health**

The Alliance for Transformative Action on Climate and Health (ATACH) has reached a major milestone, with Tuvalu becoming its 100th member. Alongside recent additions of Malaysia and the Cook Islands, the Alliance now includes 100 countries and areas dedicated to building climate-resilient, sustainable and low-carbon health systems. ATACH, a voluntary global network launched under the COP26 Health Agreement, fosters collaboration among members to share knowledge, tools and best practices for tackling the health impacts of climate change.

**Figure:**
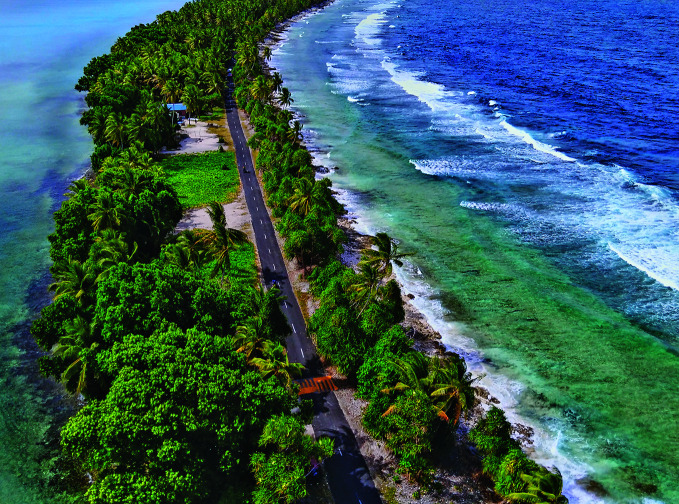
WHO/Yoshi Shimizu

## Urgent action on neurological care

The World Health Organization (WHO) has warned that neurological disorders are now among the world’s leading causes of death and disability, responsible for more than 11 million deaths annually and affecting over 3 billion people, nearly 40% of the global population.

According to WHO’s new report *Global status report on neurology*, fewer than one in three countries have a national policy to address neurological conditions such as stroke, dementia, epilepsy and migraine. Low-income countries face an acute shortage of specialists, with up to 80 times fewer neurologists than high-income nations.

“With more than one in three people in the world living with conditions affecting their brain, we must do all we can to improve the health care they need,” said Jeremy Farrar, WHO Assistant Director-General Health Promotion, Disease Prevention and Control.

WHO is urging governments to prioritize brain health through stronger policies, investment in neurological care and inclusion of these conditions in universal health coverage to close critical gaps in access and equity.

https://bit.ly/42SpCVT

## Upgraded epidemic intelligence system

WHO has launched version 2.0 of the Epidemic Intelligence from Open Sources (EIOS) system, a major upgrade designed to enhance early detection of global health threats. Developed in collaboration with international partners, the system is now used by more than 110 Member States and around 30 global organizations and networks.

Hosted at the WHO Hub for Pandemic and Epidemic Intelligence in Berlin, EIOS 2.0 integrates new data sources and artificial intelligence (AI) to improve real-time analysis of publicly available information. This enables faster identification and response to potential outbreaks.

“Today, we are not just celebrating the launch of a new version of a system, we are entering a new phase in how the world collaborates, innovates and responds to health threats,” said Dr Chikwe Ihekweazu, WHO Health Emergencies Programme Executive Director.

Building on lessons from coronavirus disease 2019 (COVID-19), mpox and avian influenza, the upgraded system aims to strengthen global preparedness and ensure rapid, coordinated action against emerging health risks.

https://bit.ly/3JcdJ6C

## New guidelines on postpartum haemorrhage

The International Federation of Gynaecology and Obstetrics (FIGO), the International Confederation of Midwives (ICM) and WHO have released landmark guidelines to transform the prevention and treatment of postpartum haemorrhage (PPH), the world’s leading cause of maternal death. PPH causes nearly 45 000 deaths annually, most of them preventable.

The new recommendations call for earlier detection and faster intervention, including a revised diagnostic threshold of 300 mL of blood loss combined with vital sign changes. The guidelines recommend the immediate deployment of the MOTIVE bundle of actions once PPH has been diagnosed: uterine massage; oxytocic drugs and tranexamic acid to stop bleeding; intravenous fluids; vaginal and genital tract examination; and escalation of care if bleeding persists.

“Women affected by PPH need care that is fast, feasible, effective and drives progress towards eliminating PPH-related deaths,” said Professor Anne Beatrice Kihara, President of FIGO. “These guidelines take a proactive approach of readiness, recognition and response. They are designed to ensure real-world impact, empowering health workers to deliver the right care, at the right time, and in a wide range of contexts.” 

Launched at the 2025 FIGO World Congress in Cape Town, the guidelines mark a major step toward ending preventable maternal deaths by improving readiness, recognition and response across all health systems.

https://bit.ly/3Jcl2LA

## The Maldives achieves triple elimination

In a historic global health milestone, WHO has validated the Maldives as the first country to eliminate mother-to-child transmission of HIV, syphilis and hepatitis B – achieving what is known as *‘triple elimination’*.

“Maldives has shown that with strong political will and sustained investment in maternal and child health, elimination of mother-to-child transmission of these deadly diseases, and the suffering they bring, is possible,” said Tedros Adhanom Ghebreyesus, WHO Director-General.

The achievement builds on the Maldives’ earlier validation for HIV and syphilis elimination in 2019. With over 95% of pregnant women receiving antenatal care and universal screening for all three infections, the country recorded zero cases of HIV and syphilis in newborns and confirmed no hepatitis B among young children in recent surveys.

Backed by universal health coverage and strong immunization systems, the Maldives’ success sets a powerful precedent for countries worldwide striving to protect mothers and infants from preventable infections.

https://bit.ly/4qDBYvo

## Global antibiotic resistance

A new WHO report reveals that one in six bacterial infections worldwide in 2023 was resistant to antibiotics, posing a growing threat to global health. Between 2018 and 2023, resistance increased in more than 40% of pathogen–antibiotic combinations, with annual rises of 5–15%.

Drawing on data from over 100 countries through the Global Antimicrobial Resistance and Use Surveillance System (GLASS), the *Global antibiotic resistance surveillance report 2025* shows that resistance is highest in the South-East Asia and Eastern Mediterranean regions, where one in three infections is untreatable with first-line antibiotics.

“Antimicrobial resistance is outpacing advances in modern medicine, threatening the health of families worldwide,” said Tedros Adhanom Ghebreyesus, WHO Director-General.

The report highlights alarming resistance among Gram-negative bacteria, including *Escherichia coli* and *Klebsiella pneumoniae*, which are responsible for severe bloodstream infections. WHO urges countries to strengthen laboratory surveillance, ensure equitable access to quality medicines and accelerate innovation in new antibiotics and rapid diagnostics to protect future generations.

https://bit.ly/47n30y6

## Digital library for traditional medicine 

WHO will launch the Traditional Medicine Global Library (TMGL) in December 2025 at the Second Global Summit on Traditional Medicine in New Delhi, India. The TMGL will be the world’s most comprehensive digital repository for traditional, complementary and integrative medicine.

Currently integrating over 1.5 million records, including evidence maps, journals, multimedia resources and national policies, the TMGL will feature six regional portals and 194 country pages, offering equitable access to diverse medical knowledge systems worldwide.

“It should be our joint effort to build a global repository for traditional medicine,” said Narendra Modi, Prime Minister of India.

The initiative supports WHO’s *Global Traditional Medicine Strategy 2025–2034*, aiming to strengthen the evidence base for traditional medicine and foster respect for Indigenous and local knowledge systems. By linking with Research4Life, the TMGL will expand global access to information, empowering communities, researchers and policy-makers to integrate traditional medicine safely and effectively.

https://bit.ly/4qpWSOo

## MERS-CoV added to BioHub 

WHO has added an isolate of the Middle East respiratory syndrome coronavirus (MERS-CoV) to its BioHub system, marking an important step in advancing research and preparedness against pathogens with pandemic potential.

WHO’s BioHub enables countries to voluntarily share and access verified biological materials linked to epidemic and pandemic threats, supporting global collaboration on surveillance, risk assessment, and the development of vaccines, diagnostics and treatments.

“Since its identification, outbreaks caused by MERS-CoV have been sporadic. As such, MERS-CoV isolates have been challenging to obtain, making it all the more important that the WHO BioHub system provides researchers with access to this virus isolate,” said Maria Van Kerkhove, acting director of WHO’s Epidemic and Pandemic Management Department.

The inclusion of this isolate expands the hub’s growing collection of pathogens and underscores WHO’s commitment to timely, equitable and secure sharing of materials to bolster global health security and pandemic preparedness.

https://bit.ly/47rKxAD

**Cover photo**

A licensed traditional medical practitioner sorts barks of trees for drying, Ghana.

**Figure:**
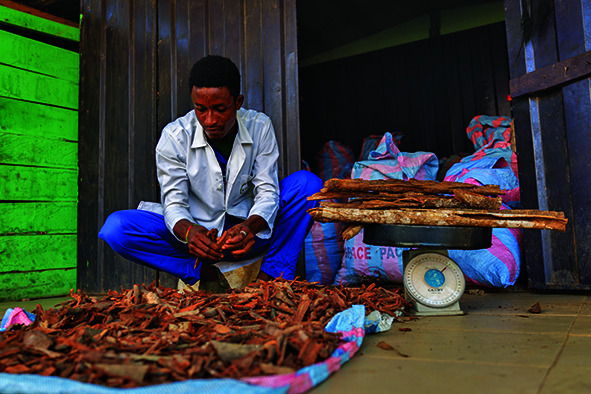
WHO/Ernest Ankomah

**Looking ahead**

14 November. World Diabetes Day. https://bit.ly/47C2n4Z

18–24 November. World AMR Awareness Week. https://bit.ly/4hpC711

17–19 December. WHO 2^nd^ Global Summit on Traditional Medicine in New Delhi, India. https://bit.ly/49o65R0

